# Supplementary material for: Competition in the chaperone-client network subordinates cell-cycle entry to growth and stress
Source: Life Sci Alliance. 2019 Apr 15;2(2):e201800277. doi: 10.26508/lsa.201800277 (PMC6467244; doi:10.26508/lsa.201800277)
Supplement: Supplementary file 6 [file LSA-2028-00277_TableS1.doc]

Supplementary Table 1. Yeast strains and plasmids.

| Yeast strains | |
| --- | --- |
| CML128 (*MAT****a*** *leu2-3,112 ura3-52 trp1-1 his4-1 can^r^*) | Gallego *et al*, 1997 |
| CML203 (*CLN3-3HA::GEN*) | Gallego *et al*, 1997 |
| CYC038 (*cln3::GEN)* | This study |
| CYC216 (*CDC28-sGFP::GEN tTA::LEU2)* | Wang *et al*, 2004 |
| CYC228 (*CDC28-sGFP::GEN cln3::LEU2*) | Wang *et al*, 2004 |
| MAG261 (*YDJ1-sGFP-FS::HIS3*) | This study |
| MAG676 (*SSA1-sGFP::HIS3*) | This study |
| MAG713 (*whi7::GEN*) | This study |
| MAG716 (*cln3::GEN whi5::NAT stb1::HYG)* | This study |
| MAG1086 (*CDC28-sGFP::GEN GAL4-ER-VP16::URA3)* | This study |
| MAG1092 (*CDC28-sGFP::GEN WHI5-mCherry::HYG*) | This study |
| MAG1512 (*TEF1_p_-mCherry::NAT*) | This study |
| MAG1533 (*CDC28-sGFP::GEN ydj1::NAT*) | This study |
| MAG1911 (*TEF1_p_-mCherry::NAT SSA1-sGFP::GEN*) | This study |
| MAG1913 (*TEF1_p_-mCherry::NAT CDC48-sGFP::GEN*) | This study |
| MAG1919 (*TEF1_p_-mCherry::NAT HSC82-sGFP::GEN*) | This study |
| KSY083-5 (*mCitrine-CLN3-11A::NAT*) | Schmoller *et al*, 2015 |
| MAG1306 (*mCitrine-CLN3-11A::NAT ydj1::GEN*) | This study |
| MAG1334 (*mCitrine-CLN3-11A::NAT HTB2-mCherry::HYG*) | This study |
| Plasmids | |
| pGEX-KG (*tac_p_-GST)* | ATCC77103 |
| pMAG85 (*tac_p_-GST-CLN3^1-75aa^*) | This study |
| pMAG87 (*tac_p_-GST-CLN3^70-220aa^*) | This study |
| pMAG89 (*tac_p_-GST-CLN3^215-320aa^*) | This study |
| pMAG91 (*tac_p_-GST-CLN3^315-420aa^*) | This study |
| pMAG93 (*tac_p_-GST-CLN3^415-580aa^*) | This study |
| pMAG155 (*tac_p_-GST-luc*) | This study |
| pMAG157 (*tac_p_-GST-P6*) | This study |
| pMAG144 (*ARS-CEN URA3 HSC82 CDC37*) | This study |
| pMAG146 (*ARS-CEN LEU2 SSA1 YDJ1*) | This study |
| pMAG149 (*ARS-CEN TRP1 CDC48 UFD1 NPL4*) | This study |
| pMAG438 (*ARS-CEN 2xTEL URA3 TRP1 HSC82 CDC37 SSA1 YDJ1 CDC48 UFD1 NPL4*) | This study |
| pMAG469 (*ARS-CEN LEU2 GAL1_p_-SSA1 GAL10_p_-YDJ1*) | This study |
| pMAG1228 (*ARS-CEN URA3 TEF1_p_-sGFP*) | This study |
| pMAG1915 (*ARS-CEN LEU2 SSA1-sGFP*) | This study |
| pMAG1917 (*ARS-CEN TRP1 CDC48-sGFP*) | This study |
| pMAG1920 (*ARS-CEN URA3 HSC82-sGFP*) | This study |
